# The effects of iTBS to cerebellar vermis on balance function in frail older people: A protocol for a randomized controlled trial

**DOI:** 10.1371/journal.pone.0339030

**Published:** 2026-01-22

**Authors:** Zuoyan Liu, Neng Huang, Kehong Zhao, Na Long, Ruibao Fu, Xiuying Hu

**Affiliations:** 1 Innovation Center of Nursing Research, Nursing Key Laboratory of Sichuan Province, West China Hospital, Sichuan University, Chengdu, China; 2 West China School of Nursing, Sichuan University, Chengdu, China; 3 Department of Rehabilitation Medicine Center, West China Hospital, Sichuan University, Chengdu, China; 4 Jinan Vocational College of Nursing, Jinan, China; National Trauma Research Institute, AUSTRALIA

## Abstract

**Background:**

With the gradual deepening of the aging of the population, the closely related problem of frailty in older people is becoming increasingly prominent. Frailty can increase the risk of bodily damage and adverse health outcomes such as falls, disabilities, hospitalizations, institutionalization, and death. Intermittent theta burst stimulation is a special stimulation mode of repetitive transcranial magnetic stimulation, which can enhance synaptic transmission by mimicking cortical theta rhythms. Some previous studies have suggested that intermittent theta burst stimulation is effective in improving balance function and motor function. However, few studies have explored the effects of intermittent theta burst stimulation to cerebellar vermis on balance function in frail older people.

**Objective:**

To introduce a randomized controlled trial protocol, which will be used to evaluate the efficacy of intermittent theta burst stimulation to cerebellar vermis on balance function in frail older people.

**Methods:**

This trial is a double-blind, two-arm randomized controlled trial. Frail older people with balance dysfunction will be recruited. All participants will be randomly assigned to experimental group who will receive intermittent theta burst stimulation to cerebellar vermis and control group who will receive false stimulation in a 1:1 ratio. All participants will also receive motor training once a day for 30–60 minutes each time, for a total of 20 sessions. The primary outcome will be balance function and the secondary outcomes will include frailty, walking ability, sensory organisation test, limits of stability, and rhythmic weight shift. All outcomes will be collected at baseline and in the week immediately post-intervention.

**Discussion:**

This study will be the first randomized controlled trial on the effects of iTBS to cerebellar vermis on balance function in frail older people. It is expected that this study, if proven effective in improving the balance function in frail older people, will provide evidence-based rehabilitation strategies for rehabilitation therapist.

**Trial status:**

Screening of participants will begin in September 2025.

## 1. Introduction

The aging of the world’s population is proceeding at an unprecedented rate, and by 2050, the population aged 60 and above is expected to increase to over 2 billion [1]. With the gradual deepening of the aging of the population, the closely related problem of frailty in older people is becoming increasingly prominent [2]. Frailty is a common condition in older people, which generally refers to a clinical syndrome characterized by a decline in functioning in multiple physiological systems [3]. The incidence of frailty among older people ranges from 4% to 59.1% in European countries [4], 6.1% to 9.0% in Japan [5], and 7.4% to 14.2% in China [6]. Frailty can increase the risk of bodily damage. Due to the loss of homeostasis, frailty may lead to a decrease in resistance to stressors and an increased risk of adverse health outcomes such as falls, disabilities, hospitalizations, institutionalization, and death [7]. These adverse consequences can seriously threaten the activities of daily living and quality of life of older people in their later years, bringing great medical and care burdens to family and society. Compared to healthy older people, the frail older people have sensory dysfunction, brain atrophy and brain dysfunction, cognitive decline, decreased muscle strength and decreased muscle mass, which may break the pathway of balance control and lead to balance dysfunction [8]. The balance dysfunction accompanied by frailty is an important factor that hinders healthy aging. It is of great significance for individuals, families and society to improve the balance function of the frail older population.

Frailty has the characteristics of systematicity, dynamism, and reversibility [9]. Targeted intervention programs may delay or even reverse the frailty of older people to a certain extent and prevent adverse health outcomes [10,11]. Balance control relies on a complete transmission pathway, that is sensory input-central integration-motor output, and is regulated through feedforward and feedback, which requires the participation and precise cooperation of various organ systems. The cerebellum plays a crucial role in balance control. The cerebellum is composed of the central vermis and bilateral enlarged cerebellar hemispheres. There are extensive fiber connections between the telencephalon, brainstem, and spinal cord, working together to regulate body balance, muscle tension and voluntary movements [12]. The cerebellar vermis plays an important role in maintaining balance and controlling movement. A previous study has found that the cerebellar vermis receives and integrates the sensations from the trunk and limbs, including various stimuli related to the formation of balance, vision and hearing, which are essential for maintaining balance, coordinating language expression, eye movement and trunk movement [13]. Balance stability correlates with cerebellar vermis volume [14]. In addition, the cerebellar vermis also participates in the anticipatory postural adjustment and compensatory postural adjustment of balance control during functional activities [15]. Patients with cerebellar vermis lesions mainly show balance dysfunction [16]. Moreover, an experiment showed that cerebellar vermis is a broad projection area of the cerebral motor cortex, and the cerebellum is closely connected to primary motor cortex, supplementary motor area, and dorsolateral prefrontal cortex through the cerebellar-thalamus-primary motor cortex circuit [17]. Although there is currently a lack of research directly using iTBS to stimulate cerebellar vermis to enhance balance function in frail older people, some studies have confirmed that iTBS to cerebellar vermis can effectively improve balance function in stroke patients and in patients with cerebellar-type multiple system atrophy [18,19]. Therefore, we can speculate that interventions targeting the cerebellar vermis may be effective in improving the balance function of frail older people.

Repetitive transcranial magnetic stimulation technology is a non-invasive brain stimulation that uses time-varying pulsed magnetic fields to act on the central nervous system, which induces current in the cortex, affects brain metabolism and nerve electrical activity, and thus regulates the structure and function of brain nerves [20]. Intermittent theta burst stimulation (iTBS) is a special stimulation mode of repetitive transcranial magnetic stimulation that can regulate neural plasticity, neurotransmitter and receptor sensitivity, cerebral blood flow, and promote the generation of brain-derived neurotrophic factor [21]. The application of iTBS in motor dysfunction after nerve injury has received widespread attention. Koch et al. [22] found that iTBS acting on the healthy cerebellar hemisphere of stroke patients can effectively improve their gait and balance function. Our previous study found that iTBS to cerebellum combined with motor training can effectively improve trunk control and balance function in patients with subacute and chronic stroke [23]. Furthermore, through functional near-infrared spectroscopy, it is suggested that iTBS to cerebellar vermis can improve healthy people’s cortical excitability of the supplementary motor area during balance tasks [24]. This points out that iTBS to cerebellar vermis can improve balance function by promoting cerebellar cortical plasticity. Therefore, the aim of this study is to introduce a randomized controlled trial protocol, which will be used to evaluate the efficacy of iTBS to cerebellar vermis on balance function in frail older people. The hypothesis is that iTBS to cerebellar vermis combined with motor training may be more effective in improving balance function of frail older people than false stimulation combined with motor training.

## 2. Methods

**Fig 1:**
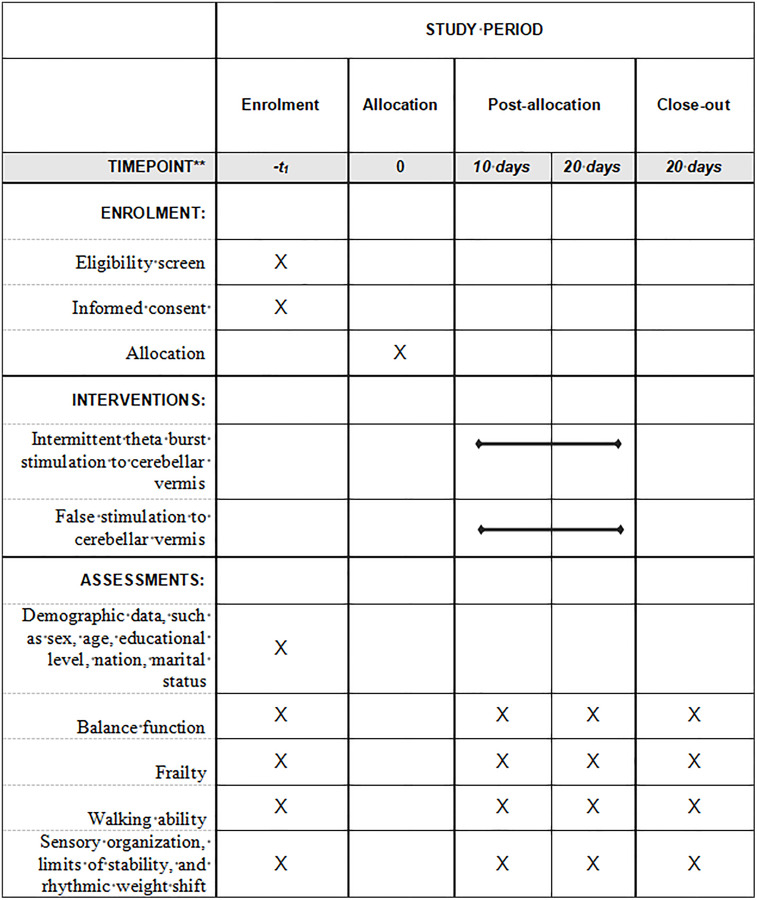
The schedule of enrolment, interventions, and assessments.

**Fig 2:**
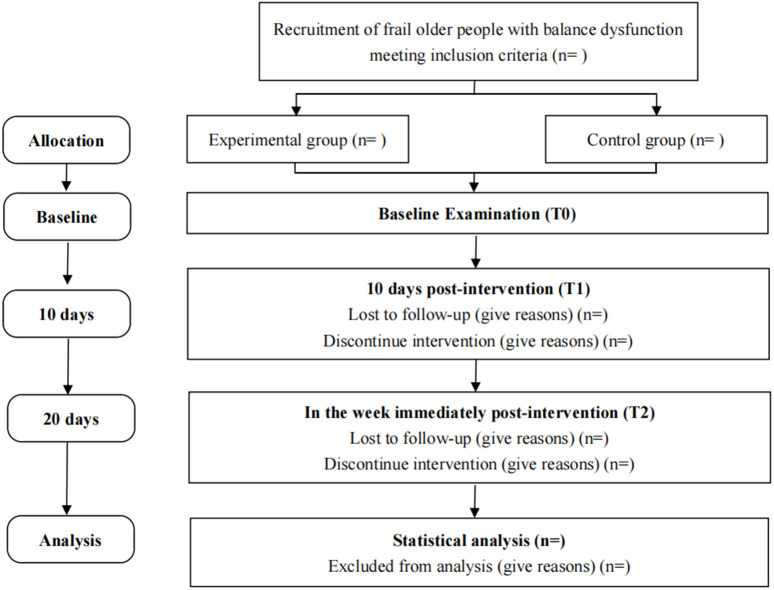
CONSORT flow diagram of this study.

This study is a single-center, double-blind, two-arm randomized controlled trial with randomization at the participant level. This trial will be carried out based on the relevant guidelines and regulations of the Declaration of Helsinki. This trial was approved by the Ethics Committee of West China Hospital of Sichuan University (number: 2024−1075) and was registered on the Chinese Clinical Trial Registry (No: ChiCTR2400082918). Written informed consent will be obtained from all participants face to face. The schedule of enrolment, interventions, and assessments is shown in Fig 1. The screening of participants will begin in September 2025. The statement of the Consolidated Standard of Reporting Trials (CONSORT) will be used to guide the report of this study [25]. This protocol was guided by the Protocol of a Clinical Trial (SPIRIT) [26]. The balance function score will be the primary endpoint for examining whether iTBS to cerebellar vermis improve balance function in frail older people. All outcomes will be collected for the two groups at baseline (T0), at 10 days post-intervention (T1), and in the week immediately post-intervention (T2). Fig 2 shows the overview of the study design.

### 2.1 Study setting and recruitment

This trial will be conducted at the department of rehabilitation medicine center of West China Hospital of Sichuan University in Chengdu, China. Researchers will recruit participants in the neurology ward. The recruitment program is planned to start in September 2025 and end in July 2027. Inclusion criteria of participants will be: aged 65 years and above; have balance dysfunction and the score of Berg Balance Scale < 56; have frailty and the score of Tilburg Frailty Indicator ≥ 5; no contraindications for iTBS, such as intracranial metal implants, cardiac pacemakers, and a history of seizures; no severe cognitive impairment and the score of Mini-Mental State Examination ≥ 17; stable vital signs and without serious heart, lung, circulation and metabolism diseases that are not suitable for motor training; voluntarily participate in this study and sign an informed consent form. The exclusion criteria will be: have other diseases except frailty that seriously affect the balance function, such as hemiplegia, paraplegia, Parkinson’s disease, lower limb fracture, spinal fracture, and amputation; participate in other clinical trials before. The dropping criteria will be: do not finish the training.

### 2.2 Sample size

G Power software (version 3.1.9.2) will be used to calculate the sample size. The balance function measured by the Berg Balance Scale will be the primary outcome. According to a previous study on stroke patients [23], the estimated effect size is 0.380. Alpha is 0.05 (bilateral) and Beta is 0.10. Correlation between repeated measurements is 0.5 and non spherical correction is 1. The total sample size is calculated to be 40. Considering dropout and loss to follow-up, the total sample size is increased by 20%, and the final total sample size is 48.

### 2.3 Randomization, allocation concealment and blinding

A 1:1 random number sequence will be generated based on a computer. After assessment at baseline, a research assistant will randomly assign eligible participants to one of two groups at a 1:1 ratio after the initial evaluation. The research assistant will place the randomized grouping schemes into some sequentially encoded, sealed, and opaque envelopes. All envelopes will be neatly stored in the locked filing cabinet of the research center in order of their entry numbers. The keys will be kept by the department secretary, who will not participate in any intervention. After evaluating the eligibility of participants and obtaining informed consent, the researchers will request the department secretary to open the envelopes in the order of enrollment to determine participants’ group. The researchers can not be blinded to the assigned interventions. Participants will be blinded to the allocation. Participants will only know that they will receive intervention when entering the group, but they will not know whether it is a real stimulus or a fake stimulus. The two intervention processes will be completely identical in operation. The data collector and statisticians will be blinded to the allocation. The data collector will only record the participant’s enrollment number during the evaluation and will not access any information related to the group. During the data entry and statistical analysis phase, the statisticians will only receive de-identified data labeled with group numbers.

### 2.4 Interventions

**Fig 3:**
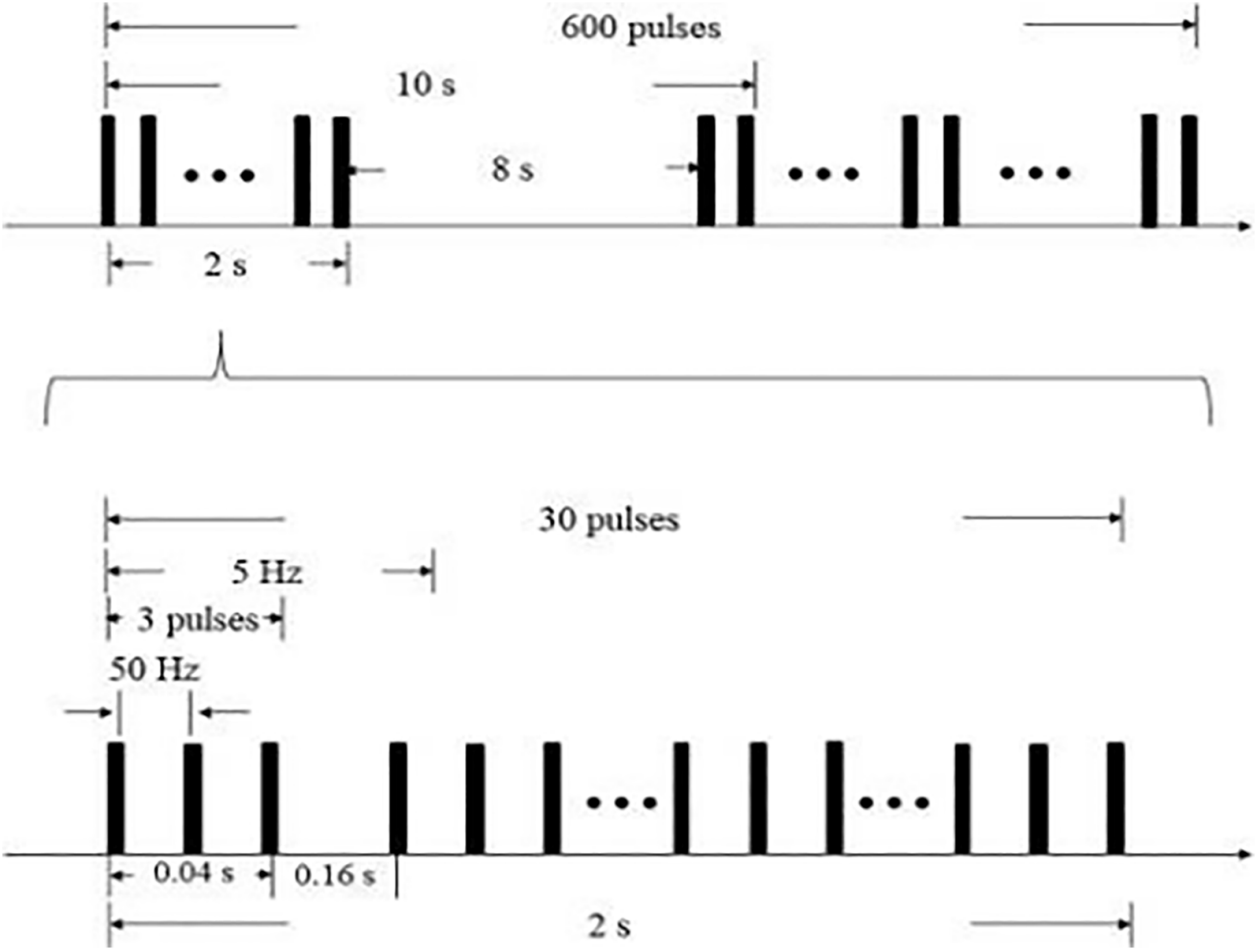
The stimulation mode of iTBS. The iTBS mode will include 600 pulses, 3 pulses per bundle, with an intra bundle frequency of 50 Hz, stimulation of 0.04s, interval of 0.16s, inter bundle frequency of 5 Hz, continuous stimulation of 10 bundles, interval of 8s, and a total stimulation duration of 200s.

All participants will be randomly assigned to the experimental group that will receive intermittent theta burst stimulation to cerebellar vermis, or control group that will receive false stimulation to cerebellar vermis. We will used a iTBS stimulator(CCY-I type, YIRUIDE GROUP, China) and an “8” coil with an inner diameter of 70 mm. The stimulation plan will follow the safety guidelines and recommendations approved by the International Congress of Clinical Neurophysiology. Stimulation intensity will be 80% of active motor threshold. If the participants can not tolerate the preset intensity, the stimulus intensity will be adjusted to the maximum intensity that the participants can tolerate. The stimulation target is cerebellar vermis that located 1 cm below the external occipital protuberance. The parameters of iTBS were 80% of the resting motor threshold and 50 Hz pulses. The resting motor threshold will be determined as the lowest intensity of stimulation that elicited a motor-evoked potential of at least 50 μV in the relaxed first dorsal interosseus muscle in a minimum of 5 of 10 trials. As shown in Fig 3, iTBS will use intermittent θ rhythm burst stimulation and the parameters will include triplet 50 Hz bursts, repeated at 5 Hz for 2 seconds on and 8 seconds off for a total of 600 pulses per session and 200 seconds. In the experimental group, the “8” coil will be tangent to the scalp of the stimulation site, and the cerebral cortex can cut the magnetic field to generate induced current for true stimulation. In the control group, the “8” coil will be perpendicular to the scalp of the stimulation site, and the cerebral cortex can not cut the magnetic field, which does not generate induced current and can not achieve the stimulation effect.

Participants in both groups will receive motor training for balance function. Professional physical therapists will design motor training programs for frail older people with balance dysfunction based on frequency, intensity, time, type, volume, and progression (FITT-VP) principles. The motor training will include muscle strength training, aerobic training, balance training, sensory training, flexibility training, and gait training. Each motor training will be conducted after the iTBS once a day. The training duration will be 30–60 minutes each time, for a total of 20 sessions.

### 2.5 Outcome assessment

Data collection will be performed by a research assistant. Demographic data will be collected at baseline (T0). All outcomes will be collected at baseline (T0), at 10 days post-intervention (T1), and in the week immediately post-intervention (T2). The primary outcome will be balance function and the secondary outcomes will include frailty, walking ability, sensory organisation test, limits of stability, and rhythmic weight shift.

#### 2.5.1 Demographic data (T0).

Participants’ demographic data will be collected at baseline, including sex, age, educational level, nation, marital status, residence, monthly income per capita of family, duration of balance dysfunction, medication, smoking, drinking, chronic disease and main caregivers.

#### 2.5.2 Balance function (T0, T1, and T2).

The Berg Balance Scale, developed in 1989 by Katherine Berg [27], will be used to evaluate balance function. This scale consists of 14 items, such as sitting alone, standing alone, transferring, turning around and picking up. It adopts Likert 4-point rating system and each item is scored from 0 (unable to complete) to 4 (completed). The total score ranges from 0 to 56, and and a higher total score indicates better balance function. The Cronbach’s α of this scale is 0.83 [28].

#### 2.5.3 Frailty (T0, T1, and T2).

The Tilburg Frailty Indicator developed by Gobbens et al. [29] will be used to evaluated frailty level. This scale has three frailty domains, including physical frailty, psychological frailty, and social frailty. It has 15 items and each item is scored using binary classification, with 1 point for “yes” and 0 point for “no”. The total score ranges from 0 to 15. A total score of 5 or above is considered frailty. A higher total score indicates more severe frailty. The Cronbach’s α of this scale is 0.73 [30].

#### 2.5.4 Walking ability (T0, T1, and T2).

Timed-Up and Go Test (TUG test), as a rapid method, was developed by Mathias et al. [31] to measure balance problems. In this study, TUG test will be used to test participants’ walking ability. The steps are: standing up from the chair; walking forward 3 meters at normal pace; turning around; walking back to the chair at normal pace; and sitting down again. The time required to complete the entire process will be recorded. The shorter the time, the better the functional walking ability.

#### 2.5.5 Sensory organisation test, limits of stability and rhythmic weight shift (T0, T1, and T2).

Balance Master system will be used to assess participants’ sensory organisation test, limits of stability and rhythmic weight shift [32]. This system was developed by Guangzhou vedo health & science co., ltd. Balance Master include Balance Manager software, a force platform, pedal, medical grade isolated power supply, a computer with Windows 7, and a color display.

Sensory organisation test can evaluate posture control during different sensory, visual, and vestibular feedback disturbances [32]. During the testing process, inaccurate interference information will be transmitted to the patient’s eyes, feet, and joints, and will be controlled through calibrated support surfaces and/or visual environment swing references. The participant is required to maintain balance to keep their centre of gravity as steady as possible. During the sensory organisation test, each trial will last for 20 seconds and will be repeated three times [33,34].

When testing the limits of stability, a real-time display of the centre of gravity position and stability limit of participants relative to the target placed at the center of the support base will be shown. Once the command is issued, participants must accurately move their centre of gravity from the center position to one of the eight targets as soon as possible (up to 8 seconds), including forwards, forwards-right, right, backwards-right, backwards, backwards-left, left and forwards-left [35].

The rhythmic weight shift will be used to evaluate participants’ ability to perform rhythmic movements of their centre of gravity from left to right (lateral) and forwards to backwards (anterior/posterior) between two targets at three different speeds (slow, medium and fast) [32]. Motion speed and direction control for each direction and speed will be measured.

### 2.6 Statistical analysis plan

We will use IBM SPSS Statistics 23 to analyze the data. P‒P diagram will be used to test the normality of the distribution of measurement data. Mean and standard deviation will be used to describe normally distributed measurement data. The independent sample t-test and a paired-sample t-test will be used for comparisons. Ranking data and non normally distributed measurement data will be described by using the median and interquartile ranges. The Mann‒Whitney U test and Wilcoxon signed rank sum test will be used for comparisons. If the sample characteristics are different in baseline, subgroup analysis will be used. We will divide the participants into different subgroups based on certain characteristics, such as age, gender and educational level, and then analysis will be conducted within each subgroup. Counting data will be described by using number and percentage. Fisher’s exact test will be used for comparisons. Repeated measures analysis of variance (group * time) will be to examine the changes in all repeated measurement outcomes. If the baseline indicators are not comparable, an adjustment to the efficacy contrast of variables will be required. The intention-to-treat principle was used as the analytical strategy for outcome evaluation. *P* < 0.05 indicates statistical significance. All tests are 2-sided.

## 3. Discussion

Frailty is an age-associated biological syndrome. With the advent of global aging, the number of frail older people is increasing and the prevalence of frailty in people older than 65 years is high [36,37]. Frailty is the main risk factor for disability [38,39]. Frail older patients frequently experience a decline in muscle strength, gait speed and balance, which ultimately leads to the loss of independence in daily activities and falls [40,41]. Therefore, it is important to improve balance function in frail older people.

It is crucial that frailty is reversible, and timely rehabilitation intervention for older people with frailty can help improve their frailty level and alleviate the adverse reactions caused by frailty. This paper outlines a protocol for the evaluation of iTBS to cerebellar vermis on balance function in frail older people with balance dysfunction. The outcomes of this randomized controlled trial will be used to provide information for clinical decision-making.

This study trials an iTBS intervention, which specifically targets cerebellar vermis that receive little attention. To the authors’ knowledge, this is the first study to examine the effects of iTBS to cerebellar vermis on balance function in frail older people. Therefore, this study represents an important contribution to the evidence base for rehabilitation intervention targeted to this underserved population. iTBS can simulate the burst discharge pattern of physiological action potentials in the central nervous system, such as the common 5 Hz cluster and bundle action potential patterns in the hippocampus [42]. It has the advantages of short stimulation time, low stimulation intensity, long action time, and being closer to the physiological state of neural activity [43], making it easier to induce neural plasticity [44]. In addition, maintaining balance depends on the complex coordination and integration of multiple systems within the body [45]. According to a fMRI research, the cerebellar vermis is strongly connected with the visual network [46]. It is also suggested that the cerebellar vermis can coordinate eye and body movements, and provide balance-related visual and auditory input [13]. Therefore, based on previous evidence, we believe that this protocol will guide the development of a meaningful clinical study and the findings will be valuable.

However, the study also has some limitations. First, this is a single-center trial. So, the findings can not represent the entire population. After completing the trial in one center, we will consider conducting multi-center validation. Moreover, it is unknown whether this research plan is accepted by the participants. So, we will conduct a feasibility trial according to this protocol to make sure the progress of the formal trial.

In conclusion, this study aims to provide a randomized controlled trial protocol, to evaluate the efficacy of intermittent theta burst stimulation to cerebellar vermis on balance function in frail older people. It may provide more rehabilitation support to frail older people with balance dysfunction and promote the realization of healthy aging.

## Supporting information

S1 File SPIRIT checklist. (DOCX)

S2 File Biomedical ethics research program. (DOC)

S3 File Protocol translated version. (DOC)
