# Supplementary material for: The effects of iTBS to cerebellar vermis on balance function in frail older people: A protocol for a randomized controlled trial
Source: PLoS One. 2026 Jan 22;21(1):e0339030. doi: 10.1371/journal.pone.0339030 (PMC12826461; doi:10.1371/journal.pone.0339030)
Supplement: S2 File — (DOC) [file pone.0339030.s002.doc]

**生物医学伦理研究方案**

**（干预性临床研究）**

小脑蚓部iTBS对衰弱老年人群的平衡功能影响及其神经调控机制研究**方案**

研究单位：四川大学华西医院

项目负责人（签名）：刘祚燕

承担科室：康复医学科

联系电话：18980606071

组长单位：四川大学华西医院

参加单位：四川大学华西医院

研究年限：2024年2月 —— 2025年4月

版本号：V2.0

版本日期：2024年7月2日

**方 案 摘 要**

| **研究设计**  **(可多选)** | **□病例对照研究 □队列研究 □横断面研究**  **☑随机对照研究 ☑应用盲法 □其他：** |
| --- | --- |
| **研究类型**  **（请根据项目类型勾选）** | **（A类：高风险）**  □Ⅲ类临床新技术（安全性、有效性确切，技术难度大、风险高）  □ 特殊人群研究（儿童、孕妇、智力低下者、精神障碍受试者等）  □ 超药物说明书研究（□超适应症 □超给药途径 □超剂量 □超年龄  □超禁忌症 □超人群 □其他，请说明： ）  □ 超器械说明书研究（□超适应症 □使用范围 □超禁忌症 □超人群  □其他，请说明： ）  □ 其他（研究者判定，请说明： ）  **（B类：中风险）**  □ 上市后生物制剂研究（预防用和治疗用）  □ 上市后治疗性疫苗研究  □ 上市后罕见病药物研究  □ Ⅱ类临床新技术（安全性、有效性确切，有一定技术难度，有一定医疗风险和伦理风险）  □ 其他（研究者判定，请说明： ）  **（C类：低风险）**  □ 已上市5年药物研究（包括化药、仿制药等）  □ 已上市器械研究（含AI，影像软件）  ☑ Ⅰ类临床新技术（安全性、有效性确切，技术难度低、几乎不存在伦理风险的医疗技术）  □ 其他（研究者判定，请说明： ） |
| **病例总数** | 48例 |
| **风险/受益分析** | 风险分析：受试者可能出现由于间歇性θ波脉冲刺激所引起的头痛、疲惫等状况，当出现以上情况，研究者会立即中止治疗，并由监护的医护人员进行护理。  受益分析：受试者将接受干预后，预期能够改善平衡和步行功能，提高运动能力。 |
| **风险判断** | □不大于最小风险 ☑大于最小风险  最小风险：指试验中预期风险的可能性和程度不大于日常生活、或进行常规体格检查或心理测试的风险 |

**1. 研究设计与方法**

本研究设计为单中心、双盲、平行随机对照临床试验，所有受试者按照1:1的比例均衡随机分配到真刺激组(24例）与伪刺激组(24例）。采用计算机随机法生成随机数字，由研究辅助人员将随机分组方案放入按顺序编码、密封不透光的信封中。由特定研究人员招募并评估受试者合格性，获取其本人及其法定监护人同意并签署知情同意书，确定入组后，请求保存分配方案的第三方按照其入组顺序拆开信封确定其分配入组情况，并独立设定其经颅磁刺激方案，再由另一组研究人员执行小脑蚓部iTBS刺激操作，两组受试者均接受运动训练干预。由经过培训的评定人员（与招募者及实施干预者不能为同一人）对纳入受试者进行基线和结局指标的评定，每位受试者治疗前后评定须由同一研究人员进行评定，评估者仅实施评估过程，不参与干预，不知晓受试者分组情况。

1. **研究对象**

在四川大学华西医院老年科及康复科招募符合标准的老年失能受试者，受试者的纳入排除标准如下：

**纳入标准：**

1. 年龄60岁及以上；
2. Berg平衡评分在21~45分之前，存在跌倒风险，但可在辅助下步行；
3. 无重度认知功能障碍，MMSE≥17分；
4. 生命体征稳定，不伴有严重的心、肺、循环、代谢等不适宜运动的严重疾病；
5. 知情同意，志愿受试，受试者本人及其监护人签署知情同意书。

**排除标准：**

1. 重度失能或存在其他严重影响患者平衡功能的疾病（如截肢、视力障碍等），导致患者在辅助下也无法步行；
2. 存在经颅磁刺激禁忌症；
3. 参加其他临床试验可能影响本试验的最终评定结果。

**剔除和脱落标准：**

1. 纳入后发现不符合标准或没有任何数据者，需予剔除；

研究期间，因各种原因退出或未完成研究规定的流程，计入脱落

**3.干预方案**

真刺激组受试者接受小脑蚓部iTBS真刺激联合运动训练；伪刺激组接受小脑蚓部iTBS伪刺激联合运动训练。

小脑蚓部iTBS刺激操作：①采用CCY-I型磁场治疗仪（武汉依瑞德公司，型号：YRD CCY-1；编号：96746749），连接内径为70mm的“8”字线圈，刺激方案严格遵守国际临床神经生理学会批准的安全指南和建议；②刺激靶点：小脑蚓部，体表定位为枕外隆突下1cm；③刺激强度：主动运动阈值（Active motor threshold, AMT）的80%——AMT是指10次刺激中至少5次诱发出波幅超过200μv的靶肌（通常为拇展短肌）运动诱发电位（Motor-evoked potential, MEP）所需要的最低刺激强度；若受试者无法耐受预设的刺激强度，刺激强度则调整为受试者所能耐受的最大强度；④ 刺激模式：iTBS间歇θ节律爆发刺激，iTBS模式包含600个脉冲，3个脉冲/丛，丛内频率为50Hz，刺激0.04s，间歇0.16s，丛间频率5Hz，连续刺激10丛，间歇8s，总刺激时长为200s，刺激模式如图3所示；⑤真刺激与伪刺激设置：真刺激组“8”字线圈与刺激部位的头皮相切，大脑皮层切割磁场，产生感应电流达到刺激作用；伪刺激组“8”字线圈与刺激部位的头皮垂直，大脑皮层未切割磁场，不产生感应电流，无法达到刺激作用。


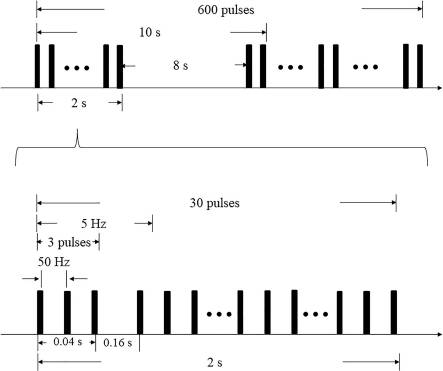


图 1 iTBS刺激模式

**运动训练：**针对老年失能人群平衡功能障碍，由专业物理治疗师为受试者设计、指导训练方案，遵循FITTVP原则。包括肌力训练、有氧训练、平衡训练、感觉训练、柔韧性训练及步态训练等，在iTBS刺激结束后立即进行训练，训练时长为30~60min，共进行20次训练。

4. 检测项目与检测时点

2组患者分别于入组时（T0）、干预结束时（20天,T1）由1位经验丰富，对分组不知情且不参与干预的物理治疗师进行评估。

结局指标：（1）临床功能评价指标：Berg平衡功能评定量表、MBI评分和通过Balance Master系统评定的平衡功能；（2）多模态脑功能监测：结构像(sMRI)、弥散张量成像(DTI)、静息功能磁共振成像(rsfMRI)、功能性近红外扫描技术（fNIRS）；（3）安全性评价：记录iTBS刺激的不良反应，如头晕、头痛、耳鸣、癫痫发作等。

5.多模态脑功能监测：

使用四川大学华西医院磁共振成像中心3.0T MRI scanner（Signa Premier, GE Healthcare, Milwaukee, USA）扫描仪对受试者进行sMRI、DTI和rsfMRI扫描。扫描参数如下：

结构像(sMRI)：3D矢状位高分辨率T1加权成像(T1-Weighted Imaging,T1WI)行结构像扫描。采用 3D SPGR 序列，重复时间(Time of Repetition, TR)=6ms, 回波时间 (Time of Echoing, TE)=1.984ms, 翻转角=9°，层厚1mm，层间距0，视野(Field of View , FOV) =256mm×256mm，矩阵(Matrix)=256×256，层数：152层。

弥散张量成像(DTI)：采用single-shot EPI序列，具体扫描参数为：TR=9000ms，TE=79.7ms，NEX=1，FOV=256mm×256mm，Matrix=128×128，体素大小=2mm×2mm×2mm，层厚=2mm，B 值=1000，包含64个梯度方向。

静息功能磁共振成像(rsfMRI)：采用快速梯度回波 EPI，TR=2000ms, TE= 30ms，层厚 4mm，翻转角=90º，FOV=240mm×240mm，Matrix=64×64，全脑35层，一共扫描255个全脑图像。

功能性近红外扫描技术（fNIRS）：使用多通道fNIRS系统（NirScan，慧创）记录SMA和DLPFC皮层中HbO_2_的变化。波长被设置为730和850nm。以10Hz的频率对数据进行采样。建立了55个通道（为相应光源-检测器对的中点），其中20个光源和20个检测器用于测量。这些通道对称分布在受试者的左右大脑半球。根据10/20国际系统，中间探针组排的中心放置在大约FPz处。光极位于左DLPFC和右DLPFC（L-DLPFC:S10-D4、S10-D9、S11-D23和S14-D23；R-DLPFC:S8-D2、S8-D8、S13-D17和S13-D20）以及左SMA和右SMA（L-SMA:S14-D15和S15-D15；R-SMA:S12-D14和S12-D20）上方。

6.不良事件的观察、记录和处置

治疗过程中，受试者如出现头晕、头痛、耳鸣等不良事件或者其他意外状况，将由医生以及责任物理治疗师进行相应的对症处理并对其发生时间、持续时间、严重程度进行相应的记录，同时根据纳入/排除标准对受试者进行重新评估以判断其是否继续研究。受试者出现头晕、头疼、耳鸣等不良反应，患者在干预后2到3小时就会缓解，无需特殊处理；若受试者干预后，持续出现头晕头疼等不良反应，将终止干预；若患者出现头晕头疼等不良反应2到3小时未缓解，给予对症处理。

7.研究的质量控制与质量保证

试验设计合理，在受试者纳入、试验干预、结局指标评估和跟踪随访方面不存在设计缺陷和实施难点；且双盲平行随机对照试验能有效避免研究结果的偏倚，具有较高的论证强度，能形成高等级研究证据。rTMS为临床上成熟应用的无创神经调控技术，iTBS是其中一种特殊的刺激模式，该治疗技术操作简便高效且几乎无严重不良反应和副作用，可保证治疗实施的同一性和安全性。脑功能多模态监测设备（sMRI, DTI, resfMRI, fNIRS）为临床上广泛应用的成熟检查设备，均为无创操作，且本研究团队成员在前期的研究中已熟练掌握相应设备的正确操作方式，保证监测结果的准确性和完整性。

四川大学华西医院康复医学中心已配备平衡大师等检测仪器；经颅磁刺激已广泛应用于脑卒中患者的功能恢复，技术和方法可行；康复医学中心与医院医学影像科存在广泛合作交流，可为本研究提供sMRI、DTI、rs-fMRI、fNIRS支持；康复医学科人员具备良好的康复基础知识以及相应的康复评定量表知识，研究所涉及的评估量表和设备均已齐备，可以为试验服务。

课题组有良好的前期研究基础和大量相关研究的工作积累。研究团队成员均已经过训且熟练掌握经颅磁刺激的操作，康复中心内有专职进行平衡大师的治疗师，保证了经颅磁刺激的准确性及结果测量的可靠性。为避免受试者脱落和保证受试者的依从性，首先，课题组会给受试者讲解本项目研究目的，受试者将获得的益处和干预中可能出现不良反应，受试者自愿且保证全程参加此项目；其次，课题组将指定成员随时跟踪受试者干预情况，以及复评、复测时间安排；最后，课题组将为每位受试者制定病例报告表。

8.数据安全监查

临床研究将根据风险大小制定相应的数据安全监察计划。所有不良事件均详细记录，恰当处理并追踪直到妥善解决或病情稳定，按照规定及时向伦理审查委员会、主管部门、申办者和药品监督管理部门报告严重不良事件与非预期事件等；主要研究者定期对所有不良事件进行累积性回顾，必要时召开研究者会议评估研究的风险与受益；双盲试验必要时可以进行紧急揭盲，以确保受试者安全与权益。

9.统计学处理

样本量计算：采用G Power（3.1.9.2）软件进行样本量计算。Berg平衡量表作为主要结局指标，根据本团队前期的试验结果（Liao，2021），估计效应量f=0.380。设置α=0.05（双尾），β=0.10，重复测量之间的相关性=0.5，非球形校正ε=1。计算得出所需样本量为40人，考虑到脱落、失访等因素，样本量增加20%，故最终总样本量为48人。

基线数据及量表评估数据：所有数据采用SPSS23.0统计软件进行统计分析。对计量资料进行正态性检验，符合正态分布的以x±s表示，组间均数比较采用独立样本t检验。计数资料以频数表示，组间比较采用χ^2^检验。等级资料或不符合正态分布的资料以中位数和四分位间距（interquartile ranges, IQRs）表示，组间比较采用Wilcoxon秩和检验。P＜0.05为差异有统计学意义。采用（组别）×（时间）的重复测量方差分析来考察干预前后及追踪时Berg平衡评分的变化情况，如果组别、时间的主效应以及组别和时间的交互作用显著的话，则进一步采取单独效应分析各个因素对因变量的影响。

sMRI 数据预处理及统计分析：①图像预处理：主要使用SPM8软件，采用基于体素的形态学分析(Voxel-Based Morphometry, VBM)方法。步骤包括图像质量检查、对准到前联合、分割成灰质、白质和脑脊液；基于DARTEL进行配准、标准化和图像调制；采用8mm半高宽高斯核平滑等。②统计分析：将每个体素的灰质体积与Berg平衡评分进行相关分析，考察小脑蚓部iTBS干预调控的相关脑区；再通过中介分析，获得介导iTBS对平衡功能影响的相关脑区。

DTI 数据预处理及统计分析：①图像预处理：DTI数据采用PANDA软件处理，具体包括图像裁剪、剥除头皮、头动及涡流校正、计算个体各向异性分数(Fractional Anisotropy, FA)及平均弥散率(Mean Diffusivity, MD)。②统计分析：基于白质骨架的弥散统计分析(Tract-Based Spatial Statistics, TBSS)，发掘自发整合怀旧与局部脑区参数的相关性；通过中介分析，获得小脑蚓部iTBS通过哪些白质纤维束的FA/MD影响平衡功能。

rsfMRI 数据预处理及统计分析：①图像预处理：主要使用DPABI软件。具体包括：去除前4个时间点的数据，时间校正、头动校正、空间标准化、平滑、滤波、去除线性漂移等。②统计分析：计算大脑局部静息参数，如低频振幅(Amplitude of Low-Frequency Fluctuation, ALFF）及局部一致性(Regional Homogeneity, ReHo)，并与Berg平衡评分进行相关分析，获取参与平衡调控的相关脑区；将ALFF、ReHo与平衡功能相关显著区域作为种子点，做全脑静息态功能连接(Resting-State Functional Connectivity, RSFC)，再将功能连接显著区域的自发神经活动结果与Berg平衡评分进行相关分析，获取显著脑区；使用中介分析，检验iTBS通过哪些脑区ALFF、ReHo及脑区间的RSFC影响平衡功能症状。

fNIRS数据预处理及统计分析：①利用NIRS-SPM工具包对fNIRS数据进行格式转换、空间定位、滤波、去漂移、一阶分析、计算beta值、组分析、绘制激活图；②利用Homer2工具包对fNIRS数据进行预处理，包括数据格式转换、伪迹检查与校正、滤波、块平均等；绘制氧合血红蛋白浓度、脱氧血红蛋白浓度以及总体血红蛋白浓度曲线图，提取峰幅度、平均幅度和达峰时间等多种特征参数；③利用Homer2对fNIRS数据进行预处理，预处理后的数据用于计算如下功能连接指标：皮尔森相关系数、（coherence）、基于相位的功能（如PLV、PLI等）、格兰杰（GCA）等；对计算的功能连接进行统计分析、多重比较校正以及功能连接可视化。

**Biomedical Ethics Research Program**

**(Interventional Clinical Study)**

The effects of iTBS to cerebellar vermis on balance function in frail older people and its neural regulation mechanisms

Institution: West China Hospital of Sichuan University

Project Leader: Liu Zuoyan

Department: Rehabilitation Medicine Department

Contact number: +8618980606071

Leader Unit: West China Hospital of Sichuan University

Participating Unit: West China Hospital of Sichuan University

Research duration: February 1, 2024 to April 30, 2025

Version number: V2.0

Version Date: July 2, 2024

**Plan abstract**

| **Research design** | **□case-control study □cohort study □cross-sectional study**  **☑Randomized controlled study ☑Application of blind method □Other：** |
| --- | --- |
| Study type | （A type：High rish）  □Ⅲ clinical new technology（with precise safety and effectiveness, high technical difficulty, and high risk）  □ Research on special populations (children, pregnant women, individuals with intellectual disabilities, subjects with mental disorders, etc.)  □ Research on super drug instructions (□ super indication □ super administration route □ super dose □ super age □ Contraindications □ Superhuman group □ Other, please specify: )  □Research on the instructions for super devices (☑ Super indications □ Scope of use □ Super contraindications □ Super population □ Other, please specify: )  □ Other (as determined by the researcher, please specify:  （B type：Medium risk）  □ Research on post market biologics (preventive and therapeutic)  □ Research on therapeutic vaccines after market launch  □ Research on Rare Disease Drugs after Listing  □ Ⅱ clinical new technology (with precise safety and efficacy, certain technical difficulties, medical and ethical risks)  □ Other (as determined by the researcher, please specify: )  （C type：Low risk）  □ Research on drugs that have been on the market for 5 years (including chemical drugs, generic drugs, etc.)  □ Research on marketed devices (including AI and imaging software)  ☑ Ⅰ clinical new technology (medical technology with precise safety and effectiveness, low technical difficulty, and almost no ethical risks)  □ Other (as determined by the researcher, please specify: ) |
| **Total cases** | 48 cases |
| **Risk/Benefit Analysis** | Risk analysis: Participants may experience headaches, fatigue, and other conditions caused by intermittent theta wave pulse stimulation. When these conditions occur, the researcher will immediately discontinue treatment and have medical staff monitor and provide care.  Benefit analysis: After the intervention, the subjects are expected to improve their balance and walking function, as well as enhance their exercise ability. |
| **Risk judgment** | □ Not greater than minimum risk ☑ Greater than minimum risk  Minimum risk: refers to the possibility and degree of expected risk in the experiment not exceeding the risk of daily life, routine physical examination or psychological testing |

Research Design and Methods

1. Design

This study is designed as a single center, double-blind, parallel randomized controlled clinical trial. All participants were randomly assigned to a true stimulation group (24 cases) and a pseudo stimulation group (24 cases) in a 1:1 ratio. Using computer randomization to generate random numbers, research assistants will place the random grouping scheme into sequentially encoded, sealed, and opaque envelopes. Recruiting and evaluating the eligibility of participants by specific researchers, obtaining their consent and that of their legal guardians, and signing an informed consent form, after determining their enrollment, requesting a third party to save the allocation plan to open the envelope according to their enrollment order to determine their allocation status, and independently setting their transcranial magnetic stimulation plan. Then, another group of researchers will perform iTBS stimulation on the cerebellar vermis, and both groups of participants will receive exercise training intervention. Trained assessors (who cannot be the same person as the recruiter or intervention provider) evaluate the baseline and outcome indicators of the included subjects. Each subject's pre - and post-treatment evaluation must be conducted by the same researcher, who only conducts the evaluation process and does not participate in the intervention, and is unaware of the subject grouping.

2. Participants

Eligible elderly disabled subjects in the Department of Geriatrics and Rehabilitation at West China Hospital of Sichuan University will be recruited.

Inclusion Criteria: Age 60 years and above; Berg balance scores between 21 and 45 indicate a risk of falling, but walking with assistance is possible; No severe cognitive impairment, MMSE score ≥ 17; Vital signs are stable and not accompanied by serious diseases such as heart, lung, circulation, metabolism, etc. that are not suitable for exercise; Informed consent, voluntary subjects, subjects themselves and their guardians sign the informed consent form.

Exclusion criteria: Severe disability or other diseases that seriously affect the patient's balance function (such as amputation, visual impairment, etc.), resulting in the patient being unable to walk even with assistance; there are contraindications for transcranial magnetic stimulation; participating in other clinical trials may affect the final evaluation results of this trial.

Dropout criteria: those found to be non compliant or without any data after inclusion shall be excluded; during the research period, if one withdraws or fails to complete the research procedures due to various reasons, it will be counted as dropout.

1. Intervention plan

The subjects in the true stimulation group received iTBS true stimulation combined with motor training in the vermis of the cerebellum; The sham stimulation group received iTBS sham stimulation combined with motor training in the vermis of the cerebellum.

ITBS stimulation operation of cerebellar vermis: ① CCY-I magnetic field therapy device (Wuhan Yiruide Company, model: YRD CCY-1; Number: 96746749), connected to an "8" coil with an inner diameter of 70mm, the stimulation protocol strictly follows the safety guidelines and recommendations approved by the International Society of Clinical Neurophysiology; ② Stimulus target: the vermis of the cerebellum, located 1cm below the occipital protuberance on the surface of the body; ③ Stimulus intensity: 80% of the Active motor threshold (AMT) - AMT refers to the minimum stimulus intensity required to induce a motor evoked potential (MEP) in the target muscle (usually the abductor pollicis muscle) with an amplitude exceeding 200 μ V in at least 5 out of 10 stimuli; If the subject cannot tolerate the preset stimulus intensity, the stimulus intensity will be adjusted to the maximum intensity that the subject can tolerate; ④ Stimulation mode: iTBS intermittent θ rhythm burst stimulation, iTBS mode includes 600 pulses, 3 pulses/bundle, intra bundle frequency of 50Hz, stimulation of 0.04s, interval of 0.16s, inter bundle frequency of 5Hz, continuous stimulation of 10 bundles, interval of 8s, total stimulation duration of 200s, as shown in Figure 1; ⑤ True stimulation and pseudo stimulation settings: In the true stimulation group, the "8" coil is tangent to the scalp of the stimulation site, and the cerebral cortex cuts the magnetic field to generate induced current for stimulation; The "8" coil of the pseudo stimulation group is perpendicular to the scalp of the stimulation site, and the cerebral cortex is not cut by the magnetic field, which does not generate induced current and cannot achieve the stimulation effect.


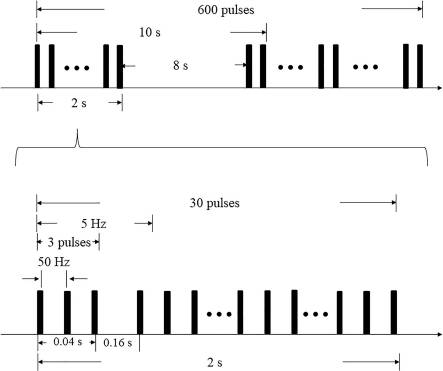


Figure 1. Stimulus pattern

Sports training: For the elderly disabled population with balance dysfunction, a professional physical therapist designs and guides a training program for the subjects, following the FITTVP principle. Including muscle strength training, aerobic training, balance training, sensory training, flexibility training, and gait training, training will be conducted immediately after iTBS stimulation, with a duration of 30-60 minutes and a total of 20 training sessions.

4. Testing items and testing time points

Two groups of patients will be evaluated by an experienced physical therapist who was unaware of the group and did not participate in the intervention at the time of enrollment (T0) and at the end of the intervention (20 days, T1).

Outcome measures: (1) Clinical functional evaluation indicators: Berg Balance Function Rating Scale, MBI score, and balance function assessed through the Balance Master system; (2) Multi modal brain function monitoring: structural imaging (sMRI), diffusion tensor imaging (DTI), resting functional magnetic resonance imaging (rsfMRI), functional near-infrared scanning technology (fNIRS); (3) Safety evaluation: Record adverse reactions of iTBS stimulation, such as dizziness, headache, tinnitus, epileptic seizures, etc.

5. Multi modal brain function monitoring

Use a 3.0T MRI scanner (Signa Premier, GE Healthcare, Milwaukee, USA) from West China Hospital of Sichuan University to perform sMRI, DTI, and rsfMRI scans on the subjects. The scanning parameters are as follows:

Structural MRI: 3D sagittal high-resolution T1 weighted imaging (T1WI) is used to scan structural images. Using a 3D SPGR sequence with a Time of Repetition (TR) of 6ms, Time of Echoing (TE)=1.984ms, Flip angle=9 °, layer thickness 1mm, layer spacing 0, Field of View (FOV)=256mm × 256mm, Matrix=256 × 256, 152 layers.

Diffusion Tensor Imaging (DTI): Single shot EPI sequence is used, with specific scanning parameters of TR=9000ms, TE=79.7ms, NEX=1, FOV=256mm × 256mm, Matrix=128 × 128, voxel size=2mm × 2mm × 2mm, slice thickness=2mm, B-value=1000, containing 64 gradient directions.

Resting functional magnetic resonance imaging (rsfMRI): using fast gradient echo EPI, TR=2000ms, TE= 30ms， Layer thickness 4mm, flip angle=90 º, FOV=240mm × 240mm, Matrix=64 × 64, 35 layers of the whole brain, a total of 255 whole brain images were scanned.

Functional near-infrared scanning technology (fNIRS): Use a multi-channel fNIRS system (NirScan, Huichuang) to record changes in HbO2 in the SMA and DLPFC cortex. The wavelengths are set to 730 and 850 nm. Sample the data at a frequency of 10Hz. 55 channels were established (as the midpoint of the corresponding light source detector pairs), with 20 light sources and 20 detectors used for measurement. These channels are symmetrically distributed in the left and right hemispheres of the subject's brain. According to the 10/20 international system, the center of the intermediate probe array is placed at approximately FPz. The light poles are located in the left DLPFC and right DLPFC (L-DLPFC: S10-D4, S10-D9, S11-D23, and S14-D23; R-DLPFC: S8-D2, S8-D8, S13-D17, and S13-D20) and left SMA and right SMA (L-SMA: S14-D15 and S15-D15; Above R-SMA: S12-D14 and S12-D20.

6. Observation, recording, and handling of adverse events

During the treatment process, if the subject experiences adverse events such as dizziness, headache, tinnitus, or other unexpected conditions, the doctor and responsible physical therapist will provide corresponding symptomatic treatment and record the occurrence time, duration, and severity. At the same time, the subject will be re evaluated according to inclusion/exclusion criteria to determine whether to continue the study. Adverse reactions such as dizziness, headache, and tinnitus may occur in the subjects, which will be relieved within 2 to 3 hours after intervention and do not require special treatment; If the subject continues to experience adverse reactions such as dizziness and headache after intervention, the intervention will be terminated; If the patient experiences adverse reactions such as dizziness and headache that do not improve within 2 to 3 hours, symptomatic treatment should be given.

7. Quality Control and Quality Assurance in Research

The experimental design is reasonable, and there are no design flaws or implementation difficulties in subject inclusion, experimental intervention, outcome indicator evaluation, and follow-up; Moreover, double-blind parallel randomized controlled trials can effectively avoid bias in research results, have high argumentative strength, and can form high-level research evidence. RTMS is a non-invasive neural regulation technique that has been maturely applied in clinical practice, and iTBS is one of its special stimulation modes. This treatment technique is simple, efficient, and has almost no serious adverse reactions or side effects, ensuring the consistency and safety of treatment implementation. The multimodal monitoring equipment for brain function (sMRI, DTI, resfMRI, fNIRS) is a mature examination device widely used in clinical practice, all of which are non-invasive operations. The members of our research team have proficiently mastered the correct operation of the corresponding equipment in previous studies, ensuring the accuracy and completeness of the monitoring results.

The Rehabilitation Medicine Center of West China Hospital of Sichuan University has been equipped with testing instruments such as Balance Master; Transcranial magnetic stimulation has been widely used for functional recovery in stroke patients, and the technology and methods are feasible; There is extensive cooperation and exchange between the Rehabilitation Medicine Center and the Medical Imaging Department of the hospital, which can provide sMRI, DTI, rs fMRI, and fNIRS support for this study; Rehabilitation medicine personnel have a good foundation in rehabilitation and corresponding knowledge of rehabilitation assessment scales. The assessment scales and equipment involved in the research are all complete and can provide experimental services.

The research team has a solid foundation in preliminary research and a wealth of accumulated work in related studies. All members of the research team have received training and are proficient in the operation of transcranial magnetic stimulation. There are dedicated therapists in the rehabilitation center who provide balance masters, ensuring the accuracy of transcranial magnetic stimulation and the reliability of result measurement. To avoid subject dropout and ensure subject compliance, firstly, the research team will explain to the subjects the purpose of this project, the benefits they will receive, and the possible adverse reactions that may occur during the intervention. The subjects voluntarily and guarantee to participate in this project throughout the entire process; Secondly, the research team will designate members to track the intervention status of the subjects at any time, as well as schedule the re evaluation and re testing time; Finally, the research team will develop a case report form for each participant.

8. Data security monitoring

Clinical research will develop corresponding data security monitoring plans based on the level of risk. All adverse events are recorded in detail, handled appropriately, and tracked until they are properly resolved or the condition stabilizes. Serious adverse events and unexpected events are promptly reported to the ethics review committee, regulatory authorities, sponsors, and drug regulatory authorities in accordance with regulations; The main researchers regularly conduct cumulative reviews of all adverse events and, if necessary, convene researcher meetings to assess the risks and benefits of the study; When necessary, emergency unblinding can be performed in double-blind trials to ensure the safety and rights of participants.

1. Statistical plan

Sample size calculation: Use G Power (3.1.9.2) software for sample size calculation. The Berg Balance Scale was used as the primary outcome measure, and based on our team's previous experimental results (Liao, 2021), the estimated effect size f was 0.380. Set α=0.05 (dual tailed), β=0.10， The correlation between repeated measurements is 0.5, and the non spherical correction ε is 1. The required sample size was calculated to be 40 people. Considering factors such as dropout and loss to follow-up, the sample size increased by 20%, resulting in a final total sample size of 48 people.

Baseline data and scale evaluation data: All data were analyzed using SPSS23.0 statistical software. Normality test shall be conducted on the measurement data, and those that conform to normal distribution shall be represented by x ± s. Independent sample t-test shall be used for inter group mean comparison. Count data is presented in frequency, and comparison between groups is conducted using a 2-test. Grade data or data that do not conform to normal distribution are represented by median and interquartile ranges (IQRs), and Wilcoxon rank sum test is used for inter group comparison. P<0.05 indicates a statistically significant difference. A repeated measures analysis of variance (ANOVA) using (group) x (time) was used to examine the changes in Berg equilibrium scores before and after intervention, as well as during tracking. If the main effects of group and time, as well as the interaction between group and time, were significant, further individual effects analysis was conducted to investigate the impact of each factor on the dependent variable.

SMRI data preprocessing and statistical analysis: ① Image preprocessing: SPM8 software is mainly used, and voxel based morphometry (VBM) method is adopted. The steps include image quality inspection, alignment to anterior commissure, segmentation into gray matter, white matter, and cerebrospinal fluid; Registration, standardization, and image modulation based on DARTEL; Using 8mm half width Gaussian kernel smoothing, etc Statistical analysis: Correlation analysis was conducted between the gray matter volume of each voxel and the Berg equilibrium score to investigate the relevant brain regions regulated by iTBS intervention in the vermis of the cerebellum; Further mediation analysis was conducted to identify the relevant brain regions that mediate the impact of iTBS on balance function.

DTI data preprocessing and statistical analysis: ① Image preprocessing: DTI data is processed using PANDA software, including image cropping, scalp removal, head movement and eddy current correction, calculation of individual fractional anisotropy (FA) and mean diffusivity (MD). ② Statistical analysis: Based on white matter skeleton diffusion statistical analysis (TBSS), explore the correlation between spontaneous integration of nostalgia and local brain parameters; Through mediation analysis, determine which white matter fiber bundles FA/MD affect balance function in the cerebellar vermis iTBS.

RsfMRI data preprocessing and statistical analysis: ① Image preprocessing: mainly using DPABI software. Specifically, it includes: removing data from the first four time points, time correction, head movement correction, spatial standardization, smoothing, filtering, removing linear drift, etc Statistical analysis: Calculate local resting parameters of the brain, such as amplitude of low frequency fluctuation (ALFF) and regional homogeneity (ReHo), and perform correlation analysis with Berg balance score to obtain relevant brain regions involved in balance regulation; Using ALFF, ReHo, and significant areas related to balance function as seed points, Resting State Functional Connectivity (RSFC) was performed in the whole brain. The spontaneous neural activity results of the significantly connected areas were then correlated with the Berg balance score to obtain significant brain regions; Using mediation analysis, examine which brain regions ALFF, ReHo, and brain regions RSFC affect balance function symptoms through iTBS.

FNIRS data preprocessing and statistical analysis: ① Use the NIRS-SPM toolkit to perform format conversion, spatial localization, filtering, drift removal, first-order analysis, beta value calculation, group analysis, and activation graph drawing on fNIRS data; ② Preprocess fNIRS data using the Homer2 toolkit, including data format conversion, artifact detection and correction, filtering, block averaging, etc; Draw curves of oxygenated hemoglobin concentration, deoxygenated hemoglobin concentration, and overall hemoglobin concentration, and extract various characteristic parameters such as peak amplitude, average amplitude, and peak time; ③ Preprocess fNIRS data using Homer2, and use the preprocessed data to calculate the following functional connectivity metrics: Pearson correlation coefficient (coherence), Phase based functions (such as PLV, PLI, etc.), Granger (GCA), etc; Perform statistical analysis, multiple comparison correction, and visualization of functional connections for computation.
