# Supplementary material for: The effects of iTBS to cerebellar vermis on balance function in frail older people: A protocol for a randomized controlled trial
Source: PLoS One. 2026 Jan 22;21(1):e0339030. doi: 10.1371/journal.pone.0339030 (PMC12826461; doi:10.1371/journal.pone.0339030)
Supplement: S3 File — (DOC) [file pone.0339030.s003.doc]

**Biomedical Ethics Research Program**

**(Interventional Clinical Study)**

The effects of iTBS to cerebellar vermis on balance function in frail older people and its neural regulation mechanisms

Institution: West China Hospital of Sichuan University

Project Leader: Liu Zuoyan

Department: Rehabilitation Medicine Department

Contact number: +8618980606071

Leader Unit: West China Hospital of Sichuan University

Participating Unit: West China Hospital of Sichuan University

Research duration: February 1, 2024 to April 30, 2025

Version number: V2.0

Version Date: July 2, 2024

**Plan abstract**

| **Research design** | **□case-control study □cohort study □cross-sectional study**  **☑Randomized controlled study ☑Application of blind method □Other：** |
| --- | --- |
| Study type | （A type：High rish）  □Ⅲ clinical new technology（with precise safety and effectiveness, high technical difficulty, and high risk）  □ Research on special populations (children, pregnant women, individuals with intellectual disabilities, subjects with mental disorders, etc.)  □ Research on super drug instructions (□ super indication □ super administration route □ super dose □ super age □ Contraindications □ Superhuman group □ Other, please specify: )  □Research on the instructions for super devices (☑ Super indications □ Scope of use □ Super contraindications □ Super population □ Other, please specify: )  □ Other (as determined by the researcher, please specify:  （B type：Medium risk）  □ Research on post market biologics (preventive and therapeutic)  □ Research on therapeutic vaccines after market launch  □ Research on Rare Disease Drugs after Listing  □ Ⅱ clinical new technology (with precise safety and efficacy, certain technical difficulties, medical and ethical risks)  □ Other (as determined by the researcher, please specify: )  （C type：Low risk）  □ Research on drugs that have been on the market for 5 years (including chemical drugs, generic drugs, etc.)  □ Research on marketed devices (including AI and imaging software)  ☑ Ⅰ clinical new technology (medical technology with precise safety and effectiveness, low technical difficulty, and almost no ethical risks)  □ Other (as determined by the researcher, please specify: ) |
| **Total cases** | 48 cases |
| **Risk/Benefit Analysis** | Risk analysis: Participants may experience headaches, fatigue, and other conditions caused by intermittent theta wave pulse stimulation. When these conditions occur, the researcher will immediately discontinue treatment and have medical staff monitor and provide care.  Benefit analysis: After the intervention, the subjects are expected to improve their balance and walking function, as well as enhance their exercise ability. |
| **Risk judgment** | □ Not greater than minimum risk ☑ Greater than minimum risk  Minimum risk: refers to the possibility and degree of expected risk in the experiment not exceeding the risk of daily life, routine physical examination or psychological testing |

Research Design and Methods

1. Design

This study is designed as a single center, double-blind, parallel randomized controlled clinical trial. All participants were randomly assigned to a true stimulation group (24 cases) and a pseudo stimulation group (24 cases) in a 1:1 ratio. Using computer randomization to generate random numbers, research assistants will place the random grouping scheme into sequentially encoded, sealed, and opaque envelopes. Recruiting and evaluating the eligibility of participants by specific researchers, obtaining their consent and that of their legal guardians, and signing an informed consent form, after determining their enrollment, requesting a third party to save the allocation plan to open the envelope according to their enrollment order to determine their allocation status, and independently setting their transcranial magnetic stimulation plan. Then, another group of researchers will perform iTBS stimulation on the cerebellar vermis, and both groups of participants will receive exercise training intervention. Trained assessors (who cannot be the same person as the recruiter or intervention provider) evaluate the baseline and outcome indicators of the included subjects. Each subject's pre - and post-treatment evaluation must be conducted by the same researcher, who only conducts the evaluation process and does not participate in the intervention, and is unaware of the subject grouping.

2. Participants

Eligible elderly disabled subjects in the Department of Geriatrics and Rehabilitation at West China Hospital of Sichuan University will be recruited.

Inclusion Criteria: Age 60 years and above; Berg balance scores between 21 and 45 indicate a risk of falling, but walking with assistance is possible; No severe cognitive impairment, MMSE score ≥ 17; Vital signs are stable and not accompanied by serious diseases such as heart, lung, circulation, metabolism, etc. that are not suitable for exercise; Informed consent, voluntary subjects, subjects themselves and their guardians sign the informed consent form.

Exclusion criteria: Severe disability or other diseases that seriously affect the patient's balance function (such as amputation, visual impairment, etc.), resulting in the patient being unable to walk even with assistance; there are contraindications for transcranial magnetic stimulation; participating in other clinical trials may affect the final evaluation results of this trial.

Dropout criteria: those found to be non compliant or without any data after inclusion shall be excluded; during the research period, if one withdraws or fails to complete the research procedures due to various reasons, it will be counted as dropout.

1. Intervention plan

The subjects in the true stimulation group received iTBS true stimulation combined with motor training in the vermis of the cerebellum; The sham stimulation group received iTBS sham stimulation combined with motor training in the vermis of the cerebellum.

ITBS stimulation operation of cerebellar vermis: ① CCY-I magnetic field therapy device (Wuhan Yiruide Company, model: YRD CCY-1; Number: 96746749), connected to an "8" coil with an inner diameter of 70mm, the stimulation protocol strictly follows the safety guidelines and recommendations approved by the International Society of Clinical Neurophysiology; ② Stimulus target: the vermis of the cerebellum, located 1cm below the occipital protuberance on the surface of the body; ③ Stimulus intensity: 80% of the Active motor threshold (AMT) - AMT refers to the minimum stimulus intensity required to induce a motor evoked potential (MEP) in the target muscle (usually the abductor pollicis muscle) with an amplitude exceeding 200 μ V in at least 5 out of 10 stimuli; If the subject cannot tolerate the preset stimulus intensity, the stimulus intensity will be adjusted to the maximum intensity that the subject can tolerate; ④ Stimulation mode: iTBS intermittent θ rhythm burst stimulation, iTBS mode includes 600 pulses, 3 pulses/bundle, intra bundle frequency of 50Hz, stimulation of 0.04s, interval of 0.16s, inter bundle frequency of 5Hz, continuous stimulation of 10 bundles, interval of 8s, total stimulation duration of 200s, as shown in Figure 1; ⑤ True stimulation and pseudo stimulation settings: In the true stimulation group, the "8" coil is tangent to the scalp of the stimulation site, and the cerebral cortex cuts the magnetic field to generate induced current for stimulation; The "8" coil of the pseudo stimulation group is perpendicular to the scalp of the stimulation site, and the cerebral cortex is not cut by the magnetic field, which does not generate induced current and cannot achieve the stimulation effect.


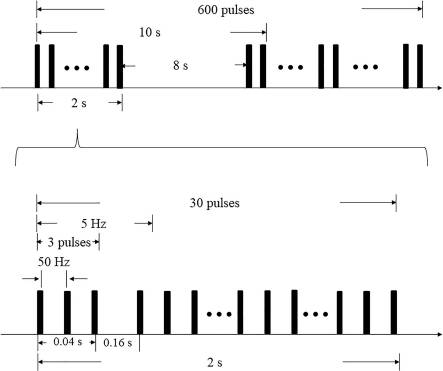


Figure 1. Stimulus pattern

Sports training: For the elderly disabled population with balance dysfunction, a professional physical therapist designs and guides a training program for the subjects, following the FITTVP principle. Including muscle strength training, aerobic training, balance training, sensory training, flexibility training, and gait training, training will be conducted immediately after iTBS stimulation, with a duration of 30-60 minutes and a total of 20 training sessions.

4. Testing items and testing time points

Two groups of patients will be evaluated by an experienced physical therapist who was unaware of the group and did not participate in the intervention at the time of enrollment (T0) and at the end of the intervention (20 days, T1).

Outcome measures: (1) Clinical functional evaluation indicators: Berg Balance Function Rating Scale, MBI score, and balance function assessed through the Balance Master system; (2) Multi modal brain function monitoring: structural imaging (sMRI), diffusion tensor imaging (DTI), resting functional magnetic resonance imaging (rsfMRI), functional near-infrared scanning technology (fNIRS); (3) Safety evaluation: Record adverse reactions of iTBS stimulation, such as dizziness, headache, tinnitus, epileptic seizures, etc.

5. Multi modal brain function monitoring

Use a 3.0T MRI scanner (Signa Premier, GE Healthcare, Milwaukee, USA) from West China Hospital of Sichuan University to perform sMRI, DTI, and rsfMRI scans on the subjects. The scanning parameters are as follows:

Structural MRI: 3D sagittal high-resolution T1 weighted imaging (T1WI) is used to scan structural images. Using a 3D SPGR sequence with a Time of Repetition (TR) of 6ms, Time of Echoing (TE)=1.984ms, Flip angle=9 °, layer thickness 1mm, layer spacing 0, Field of View (FOV)=256mm × 256mm, Matrix=256 × 256, 152 layers.

Diffusion Tensor Imaging (DTI): Single shot EPI sequence is used, with specific scanning parameters of TR=9000ms, TE=79.7ms, NEX=1, FOV=256mm × 256mm, Matrix=128 × 128, voxel size=2mm × 2mm × 2mm, slice thickness=2mm, B-value=1000, containing 64 gradient directions.

Resting functional magnetic resonance imaging (rsfMRI): using fast gradient echo EPI, TR=2000ms, TE= 30ms， Layer thickness 4mm, flip angle=90 º, FOV=240mm × 240mm, Matrix=64 × 64, 35 layers of the whole brain, a total of 255 whole brain images were scanned.

Functional near-infrared scanning technology (fNIRS): Use a multi-channel fNIRS system (NirScan, Huichuang) to record changes in HbO2 in the SMA and DLPFC cortex. The wavelengths are set to 730 and 850 nm. Sample the data at a frequency of 10Hz. 55 channels were established (as the midpoint of the corresponding light source detector pairs), with 20 light sources and 20 detectors used for measurement. These channels are symmetrically distributed in the left and right hemispheres of the subject's brain. According to the 10/20 international system, the center of the intermediate probe array is placed at approximately FPz. The light poles are located in the left DLPFC and right DLPFC (L-DLPFC: S10-D4, S10-D9, S11-D23, and S14-D23; R-DLPFC: S8-D2, S8-D8, S13-D17, and S13-D20) and left SMA and right SMA (L-SMA: S14-D15 and S15-D15; Above R-SMA: S12-D14 and S12-D20.

6. Observation, recording, and handling of adverse events

During the treatment process, if the subject experiences adverse events such as dizziness, headache, tinnitus, or other unexpected conditions, the doctor and responsible physical therapist will provide corresponding symptomatic treatment and record the occurrence time, duration, and severity. At the same time, the subject will be re evaluated according to inclusion/exclusion criteria to determine whether to continue the study. Adverse reactions such as dizziness, headache, and tinnitus may occur in the subjects, which will be relieved within 2 to 3 hours after intervention and do not require special treatment; If the subject continues to experience adverse reactions such as dizziness and headache after intervention, the intervention will be terminated; If the patient experiences adverse reactions such as dizziness and headache that do not improve within 2 to 3 hours, symptomatic treatment should be given.

7. Quality Control and Quality Assurance in Research

The experimental design is reasonable, and there are no design flaws or implementation difficulties in subject inclusion, experimental intervention, outcome indicator evaluation, and follow-up; Moreover, double-blind parallel randomized controlled trials can effectively avoid bias in research results, have high argumentative strength, and can form high-level research evidence. RTMS is a non-invasive neural regulation technique that has been maturely applied in clinical practice, and iTBS is one of its special stimulation modes. This treatment technique is simple, efficient, and has almost no serious adverse reactions or side effects, ensuring the consistency and safety of treatment implementation. The multimodal monitoring equipment for brain function (sMRI, DTI, resfMRI, fNIRS) is a mature examination device widely used in clinical practice, all of which are non-invasive operations. The members of our research team have proficiently mastered the correct operation of the corresponding equipment in previous studies, ensuring the accuracy and completeness of the monitoring results.

The Rehabilitation Medicine Center of West China Hospital of Sichuan University has been equipped with testing instruments such as Balance Master; Transcranial magnetic stimulation has been widely used for functional recovery in stroke patients, and the technology and methods are feasible; There is extensive cooperation and exchange between the Rehabilitation Medicine Center and the Medical Imaging Department of the hospital, which can provide sMRI, DTI, rs fMRI, and fNIRS support for this study; Rehabilitation medicine personnel have a good foundation in rehabilitation and corresponding knowledge of rehabilitation assessment scales. The assessment scales and equipment involved in the research are all complete and can provide experimental services.

The research team has a solid foundation in preliminary research and a wealth of accumulated work in related studies. All members of the research team have received training and are proficient in the operation of transcranial magnetic stimulation. There are dedicated therapists in the rehabilitation center who provide balance masters, ensuring the accuracy of transcranial magnetic stimulation and the reliability of result measurement. To avoid subject dropout and ensure subject compliance, firstly, the research team will explain to the subjects the purpose of this project, the benefits they will receive, and the possible adverse reactions that may occur during the intervention. The subjects voluntarily and guarantee to participate in this project throughout the entire process; Secondly, the research team will designate members to track the intervention status of the subjects at any time, as well as schedule the re evaluation and re testing time; Finally, the research team will develop a case report form for each participant.

8. Data security monitoring

Clinical research will develop corresponding data security monitoring plans based on the level of risk. All adverse events are recorded in detail, handled appropriately, and tracked until they are properly resolved or the condition stabilizes. Serious adverse events and unexpected events are promptly reported to the ethics review committee, regulatory authorities, sponsors, and drug regulatory authorities in accordance with regulations; The main researchers regularly conduct cumulative reviews of all adverse events and, if necessary, convene researcher meetings to assess the risks and benefits of the study; When necessary, emergency unblinding can be performed in double-blind trials to ensure the safety and rights of participants.

1. Statistical plan

Sample size calculation: Use G Power (3.1.9.2) software for sample size calculation. The Berg Balance Scale was used as the primary outcome measure, and based on our team's previous experimental results (Liao, 2021), the estimated effect size f was 0.380. Set α=0.05 (dual tailed), β=0.10， The correlation between repeated measurements is 0.5, and the non spherical correction ε is 1. The required sample size was calculated to be 40 people. Considering factors such as dropout and loss to follow-up, the sample size increased by 20%, resulting in a final total sample size of 48 people.

Baseline data and scale evaluation data: All data were analyzed using SPSS23.0 statistical software. Normality test shall be conducted on the measurement data, and those that conform to normal distribution shall be represented by x ± s. Independent sample t-test shall be used for inter group mean comparison. Count data is presented in frequency, and comparison between groups is conducted using a 2-test. Grade data or data that do not conform to normal distribution are represented by median and interquartile ranges (IQRs), and Wilcoxon rank sum test is used for inter group comparison. P<0.05 indicates a statistically significant difference. A repeated measures analysis of variance (ANOVA) using (group) x (time) was used to examine the changes in Berg equilibrium scores before and after intervention, as well as during tracking. If the main effects of group and time, as well as the interaction between group and time, were significant, further individual effects analysis was conducted to investigate the impact of each factor on the dependent variable.

SMRI data preprocessing and statistical analysis: ① Image preprocessing: SPM8 software is mainly used, and voxel based morphometry (VBM) method is adopted. The steps include image quality inspection, alignment to anterior commissure, segmentation into gray matter, white matter, and cerebrospinal fluid; Registration, standardization, and image modulation based on DARTEL; Using 8mm half width Gaussian kernel smoothing, etc Statistical analysis: Correlation analysis was conducted between the gray matter volume of each voxel and the Berg equilibrium score to investigate the relevant brain regions regulated by iTBS intervention in the vermis of the cerebellum; Further mediation analysis was conducted to identify the relevant brain regions that mediate the impact of iTBS on balance function.

DTI data preprocessing and statistical analysis: ① Image preprocessing: DTI data is processed using PANDA software, including image cropping, scalp removal, head movement and eddy current correction, calculation of individual fractional anisotropy (FA) and mean diffusivity (MD). ② Statistical analysis: Based on white matter skeleton diffusion statistical analysis (TBSS), explore the correlation between spontaneous integration of nostalgia and local brain parameters; Through mediation analysis, determine which white matter fiber bundles FA/MD affect balance function in the cerebellar vermis iTBS.

RsfMRI data preprocessing and statistical analysis: ① Image preprocessing: mainly using DPABI software. Specifically, it includes: removing data from the first four time points, time correction, head movement correction, spatial standardization, smoothing, filtering, removing linear drift, etc Statistical analysis: Calculate local resting parameters of the brain, such as amplitude of low frequency fluctuation (ALFF) and regional homogeneity (ReHo), and perform correlation analysis with Berg balance score to obtain relevant brain regions involved in balance regulation; Using ALFF, ReHo, and significant areas related to balance function as seed points, Resting State Functional Connectivity (RSFC) was performed in the whole brain. The spontaneous neural activity results of the significantly connected areas were then correlated with the Berg balance score to obtain significant brain regions; Using mediation analysis, examine which brain regions ALFF, ReHo, and brain regions RSFC affect balance function symptoms through iTBS.

FNIRS data preprocessing and statistical analysis: ① Use the NIRS-SPM toolkit to perform format conversion, spatial localization, filtering, drift removal, first-order analysis, beta value calculation, group analysis, and activation graph drawing on fNIRS data; ② Preprocess fNIRS data using the Homer2 toolkit, including data format conversion, artifact detection and correction, filtering, block averaging, etc; Draw curves of oxygenated hemoglobin concentration, deoxygenated hemoglobin concentration, and overall hemoglobin concentration, and extract various characteristic parameters such as peak amplitude, average amplitude, and peak time; ③ Preprocess fNIRS data using Homer2, and use the preprocessed data to calculate the following functional connectivity metrics: Pearson correlation coefficient (coherence), Phase based functions (such as PLV, PLI, etc.), Granger (GCA), etc; Perform statistical analysis, multiple comparison correction, and visualization of functional connections for computation.
